# 3D Printed Materials for Permanent Restorations in Indirect Restorative and Prosthetic Dentistry: A Critical Review of the Literature

**DOI:** 10.3390/ma17061380

**Published:** 2024-03-18

**Authors:** Dario Balestra, Morgan Lowther, Cecilia Goracci, Mauro Mandurino, Silvia Cortili, Gaetano Paolone, Chris Louca, Alessandro Vichi

**Affiliations:** 1 Dental Academy, University of Portsmouth, Portsmouth PO1 2QG, UK; dbale@uax.es (D.B.); chris.louca@port.ac.uk (C.L.); 2 School of Mechanical and Design Engineering, University of Portsmouth, Portsmouth PO1 3DJ, UK; morganlowther@gmail.com; 3 Department of Medical Biotechnologies, University of Siena, 53100 Siena, Italy; cecilia.goracci@unisi.it; 4 Dental School, IRCCS San Raffaele Hospital, Vita-Salute University, 20132 Milan, Italy; m.mandurino@studenti.unisr.it (M.M.); seuscortili@gmail.com (S.C.); paolone.gaetano@hsr.it (G.P.)

**Keywords:** 3D printing, additive manufacturing, rapid prototyping, prosthodontics

## Abstract

The objective of this study was to review the scientific evidence currently available on 3D printable materials and 3D printing technologies used for the fabrication of permanent restorations, focusing on material properties that are clinically relevant. A literature search was performed on four databases (MEDLINE/PubMed, Scopus, Cochrane Library, Web of Science) for articles published from January 2013 until November 2023, using a combination of free words: (restorative dentistry OR prosthetic dentistry) AND (3D printing OR additive manufacturing OR rapid prototyping) AND materials. Two reviewers screened titles and/or abstracts of 2.468 unique studies. In total, 83 studies were selected for full-text reading, from which 36 were included in the review. The assessed variables were mechanical properties, reporting in most of the cases positive results, dimensional accuracy and fit, reporting conflicting results with a predominance of positive, aesthetic properties, with positive reports but scarcely addressed, and biological properties, almost unexplored in independent studies. Despite numerous studies with positive results in favor, papers with negative outcomes were also retrieved. Aesthetic and biological properties are conversely still mostly unexplored. There remains a lack of conclusive evidence for viable 3D printable restorative and prosthodontic materials for permanent restorations. Research should be strengthened by defining international standards for laboratory testing and, where pre-clinical data are promising, conducting clinical trials.

## 1. Introduction

The introduction of new digital technologies related to 3D imaging, computer design, modeling, manufacturing, and material science, has deeply influenced dentistry over the past few decades [1,2,3,4,5,6,7,8].

In the earlier stages of this digital revolution, computer-aided design (CAD) and computer-aided manufacturing (CAM) were synonymous with a subtractive manufacturing process (SM), where an object is created from a block of material by milling, grinding, drilling, turning, or polishing. More recently, an additive manufacturing process (AM) based on 3D printing technology, allowing the manufacture of objects by adding and uniting successive layers of material, has been increasingly used in dentistry [9,10], especially in oral and maxillofacial surgery [11,12], scaffold production [13,14], implantology [15,16], endodontics [17,18], wearable personalized protections [19] and drug delivery [20] devices, orthodontics [21,22], and prosthodontics. Concerning the latter, the application of 3D printing to the prosthodontics workflow has enabled the digital production of removable prostheses [23,24,25], temporary and permanent crowns and fixed partial dentures [26,27,28,29,30], reducing manufacturing time and cost, and increasing versatility for the manufacturing of complex geometries.

However, achieving the full potential of 3D printing relies on improvements in both dental materials and processes [31,32,33].

The relative infancy of the application of this technology to dentistry is highlighted by conflicting results, especially concerning the mechanical properties, dimensional accuracy, and fit, of 3D printed materials for permanent restorations [26,34,35,36,37].

Other key properties require attention, such as aesthetics [38], and biocompatibility, and the latter in particular must be prioritized in prosthodontics as it represents the boundary between materials for temporary and permanent restorations. Careful consideration must be given to whether materials are for transient use, and therefore they need to satisfy Class I biocompatibility [39] or act as permanent materials and thereby require meeting the requirements for Class IIa biocompatibility due to intraoral use and long-term stability.

Given the interest in applying 3D printable materials for intraoral use, it is timely and relevant to explore the currently available scientific literature on the properties of such materials. For this purpose, a narrative review of the literature focusing on 3D printed materials for permanent restorations in indirect conservative and prosthodontic dentistry, not available at present in the dental literature, was conducted.

## 2. Materials and Methods

The literature review was conducted based on the following question: “What is the scientific evidence currently available on 3D printable materials and 3D printing technologies used for the fabrication of permanent restorations?”. The search focused on permanent restorations, and particular attention was given to the mechanical, biological, and aesthetic properties of the materials.

### 2.1. Inclusion and Exclusion Criteria

The review was restricted to the evidence on 3D printing materials for intraoral definitive use in restorative and prosthetic dentistry. Therefore, studies on materials for temporary restorations, denture bases, frameworks, metal copings, study models, surgical guides, orthodontic aligners, and indirect bonding trays were excluded from the review. In addition, publications such as reviews, editorials, comments on previous articles, and extracts from conferences were excluded.

### 2.2. Information Sources and Research Strategies

An electronic search was conducted using the following databases: MEDLINE/PubMed, Scopus, Cochrane Library, and Web of Science. The review included the latest research only in English and with full text available from January 2013 to November 2023; however, older reference manuscripts could be included. The last database consultation was performed on 23 November 2023.

Table 1 reports terms and the search strategy for each database, and the number of documents retrieved.

### 2.3. Sources of Evidence Selection

Each article was evaluated through a three-step process which consequently took into consideration the title, abstract, and full text of the manuscript. Two investigators (D.B., A.V.), working independently, judged whether each article met the inclusion criteria and was relevant to the review’s objective. In case of disagreement between the investigators, a shared decision was reached upon discussion.

By querying the databases with the defined search terms, we retrieved initially 2468 citations (MEDLINE/PubMed: *n* = 1070; Scopus: *n* = 908; Cochrane Library: *n* = 123; Web of Science: *n* = 367). After identifying and excluding duplicates, the title screening process excluded articles not evaluating materials for 3D printing, as well as those investigating printing materials for the production of temporary restorations, denture bases, frameworks, metal copings, study models, surgical guides, orthodontic aligners, and indirect bonding trays.

The abstracts of the remaining 284 articles were carefully analyzed, and 202 articles were excluded not evaluating the mechanical, biological, and aesthetic properties of the materials.

**Figure 1:**
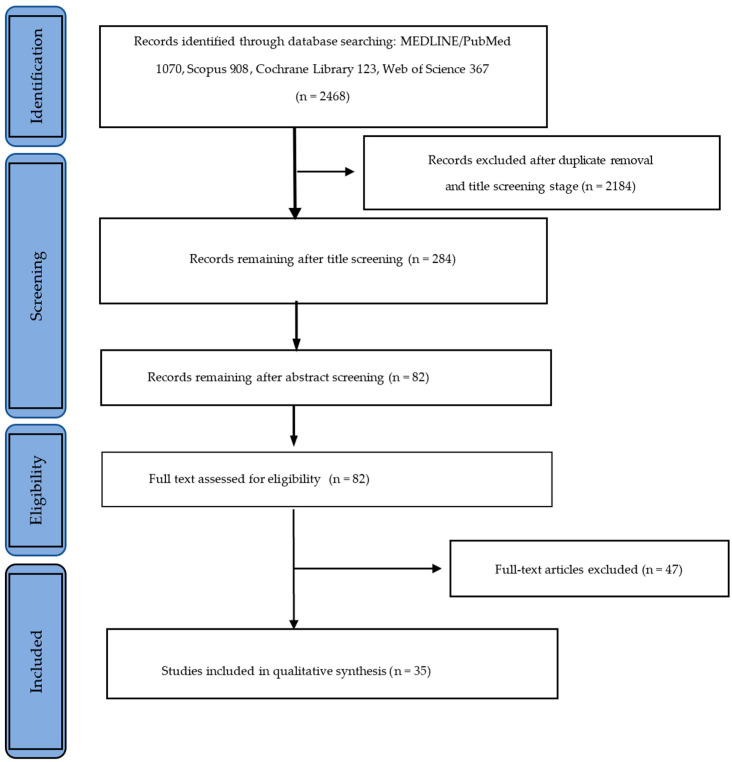
Study selection flowchart.

The full texts of the remaining 82 articles were obtained and read. Forty-seven articles were excluded from the qualitative analysis: forty did not assess permanent materials and their properties, only cited them, and seven articles were summaries of previously published literature. Figure 1 presents the study selection flowchart.

## 3. Results

The findings of the 35 studies were eventually included in the qualitative analysis and are reviewed in the following paragraphs: 1. Mechanical properties, 2. Dimensional accuracy and fit, 3. Biological properties, and 4. Aesthetic properties. Quantitative analysis was not performed in the current review. In Table 2 are reported the studies included and the assessed variables.

### 3.1. Mechanical Properties

There is much interest in evaluating the mechanical properties of 3D printing materials in comparison with the former established materials and technologies available on the market. This makes this subject currently the most investigated.

Zimmermann et al. [65] evaluated the fracture behavior of different CAD/CAM ceramics and composites (Lava Ultimate, Cerasmart, and Brilliant Crios) and one 3D printed composite (els-3D Harz, Saremco Dental AG; DLP Freeform Pro 2–ASIGA), as a function of different crown thicknesses (0.5, 1, and 1.5 mm). None of the 0.5 mm ceramic crowns survived fatigue testing, and all 0.5 mm composite crowns did. This indicates composites may have advantageous material characteristics compared to ceramic CAD/CAM materials for minimal restoration thicknesses.

In addition to 3D printed resin composites, ceramics and zirconia have also been tested [28,34,35,36,38,47,53,54,55,58].

Baumgartner et al. [38] used stereolithographic ceramic manufacturing (LCM–Powder IPS e.max Press LT, Ivoclar Vivadent AG, Schaan, Liecthenstain) to reproduce lithium disilicate glass-ceramic samples, showing the feasibility of printing dense and reliable lithium disilicate glass-ceramic samples that meet the high mechanical requirements of dental restorations. The high density of the sintered parts of >99.9% of the theoretical density indicates low porosity and leads to remarkable biaxial bending strengths of up to 430 Mpa for the samples with the highest surface quality (polished). Outstanding Weibull moduli of ≥10 show high reliability of the printing process used for these glass ceramics as well as of the thermal post-processing protocols.

Li et al. [36] evaluated the physical and mechanical properties of SL-manufactured zirconia dental crowns of a custom-made resin-based zirconia (45 vol%; CSL 150–Porimy), showing adequate results to fabricate dental crowns: density measured at 5.83 g/cm^3^, flexural strength was 812 ± 128 Mpa, Weibull modulus was 7.44.

Nakai et al. [55] evaluated the crystallography, microstructure, and flexural strength of zirconia-based ceramics made by stereolithography (SLA), with two additively manufactured 3Y-TZPs (LithaCon 3Y 230, Lithoz, Vienna, Austria; 3D Mix zirconia, 3Dceram Sinto, Limoges, France) and one additively manufactured ATZ (3D Mix ATZ, 3Dceram Sinto, Limoges, France). The results of the study showed that additively manufactured zirconia revealed a crystal structure, biaxial flexural strength, and microstructure comparable to that of subtractively manufactured zirconia. Differences in the additive manufacturing process of zirconia may affect the biaxial flexural strength of additively manufactured zirconia. Additively manufactured ATZ had a higher biaxial flexural strength than additively and subtractively manufactured 3Y-TZP.

In addition to restorations/crowns cemented on natural teeth, Zandinejad et al. [28] compared the fracture resistance of milled zirconia (MZr–LavaTM Plus Zirconia W1, 3M Co., St. Paul, MN, USA), milled lithium disilicate (MLD–IPS e.max CAD crown HT A1; Ivoclar Vivadent, Amherst, USA), and AM zirconia (AMZr–3Dmix ZrO 2paste; 3Dceram Co. Lemonge, France; CeraMaker 900; 3Dceram Co. Lemonge, France), crowns cemented to milled zirconia implant abutments (MZr). The results of the study showed that AM zirconia crowns have a comparable fracture resistance to milled zirconia crowns when cemented to zirconia abutments. MZr demonstrated the highest median fracture resistance (1292 ± 189 N), followed by MLD (1289 ± 142 N) and AMZr (1243.5 ± 265.5 N) crowns. No statistically significant differences in fracture resistance were reported between the three groups. In all three groups, the samples fractured at the abutment. The fracture line was located near the interface of the zirconia abutment and the implant analog. No significant differences were found in the mode of failure between the three groups. The crowns were intact in all groups at the end of the experimental procedure. Refaie et al. [58] evaluated the fracture resistance of 3D printed and milled zirconia crowns and found that the immediate fracture resistance of all crowns exceeded 790 N, which is higher than the physiological biting force, reported in the literature to be 450–520 N, and of the forces reported in bruxism, reported to be 790 N [66,67]. The fracture resistance was reported to be reduced after cyclic loading, thus remaining higher than 790N. Accordingly, crowns fabricated with both techniques could withstand 5 years of clinical service in the oral cavity. Miura et al. investigated the mechanical properties of additively manufactured zirconia in two different studies [53,54]. They found that flexural strength is correlated with the printing angle. A printing orientation of 90° followed by 45° resulted in the highest values of flexural strength (>500 MPa). Giugliano et al. [47] printed their specimens with an angle of 0° and found that 16.3% of their specimens did not reach the 500 MPa threshold for a three-unit prosthesis. Borella et al. [40] compared the mechanical properties of specimens 3D printed with different layer thicknesses and found that groups printed with 50 μm exhibited a higher flexural strength compared with those printed with 100 μm.

Even if several studies reported positive results in favor of the mechanical properties of 3D printed materials, some other papers reported adverse results.

Uçar et al. [35] compared the mechanical and microstructural properties of ceramics from lithography-based ceramic manufacturing (LCM), comparing 3D printed high-purity alumina (LithaLox HP 500, Lithoz; CeraFab 7500, Lithoz) with pressing and CAD/CAM methods. The studied parameters were biaxial flexural strength, hardness, fracture toughness, and structural reliability. The study demonstrated that LCM can be used to produce ceramic parts with promising mechanical properties, but improvements are needed, mainly to reduce porosity.

Revilla-León et al. [34] compared the flexural strength and Weibull characteristics of milled and additive-manufactured zirconia, using a photosensitive resin mixed with zirconia paste (3DMixZrO2L, 3DCeram Co.; CERAMAKER 900, 3DCeram Co., Bonnac-la-cote, France). The results were largely different from previous studies: AM zirconia materials revealed significantly lower flexural strength mean values than milled zirconia materials. Significantly decreased flexural strength values of milled and AM zirconia materials were indicated by the Weibull moduli being significantly higher for the milled groups than the additively manufactured groups.

Prause et al. [57] also compared the flexural strength and Weibull modulus of 3D printed composite resins with milled composite resins and PICN for definitive restorations and found that the 3D printed composite resin exhibited the lowest biaxial flexural strength. Moreover, they also observed that the fracture origin of the 3D printed composite resin was correlated with the flaws introduced by the mixing procedure taking place during the 3D printing process.

Cakmak et al. [41] compared the microhardness of additively and subtractively manufactured specimens and found that the subtractively manufactured ones had higher microhardness values.

### 3.2. Dimensional Accuracy and Fit

As dimensional accuracy and fit have great importance in clinical use, they represent another important aspect to investigate, and therefore, some relevant papers were retrieved.

Wang et al. [63] evaluated the trueness of zirconia crowns fabricated by 3D printing, using a photosensitive resin mixed with zirconia paste (3DMixZrO2L, 3DCeram Co.; CERAMAKER 900, 3DCeram Co.), in comparison with crowns fabricated by CAD-CAM milling as a control. The results showed that zirconia crowns produced by 3D printing met the trueness requirements, and 3D printing may be suitable for fabricating zirconia crowns.

Homsy et al. [48] compared the marginal and internal fit accuracy of lithium disilicate glass-ceramic inlays fabricated with conventional, milled, and 3D printed wax patterns using a polymer (VisiJet FTX Green, 3D Systems; ProJet 1200, 3D Systems). The CAD-CAM subtractive method of wax pattern fabrication produced IPS e.max Press inlays with better marginal and internal fittings than those obtained through conventional workflows or additive 3D printing. Three-dimensional printing of inlay wax patterns yielded similar results to conventional waxing in terms of marginal and internal fit.

Bae et al. [27] evaluated the accuracy of inlay restorations fabricated by AM (polymer VisiJet FTX Green, 3D Systems; ProJet 1200, 3D Systems), compared to subtractive methods. The result of the study showed that the accuracy of inlays fabricated by AM is also higher in comparison with subtractive methods.

Revilla-León et al. [59] measured the manufacturing accuracy and volumetric changes of AM zirconia specimens (3DMix ZrO_2_ paste; 3DCeram Co.; CERAMAKER 900, 3DCeram Co.) with different porosities (0%, 20%, and 40%). The results showed that the 40%-porosity group obtained the highest manufacturing accuracy and the lowest manufacturing volume change, followed by the 20%-porosity and the 0%-porosity groups. An uneven manufacturing volume change in the *x*-, *y*-, and *z*-axes was observed. However, none of the groups tested were able to perfectly match the virtual design of the specimens.

Wang et al. [29] evaluated the dimensional accuracy and clinical adaptation of ceramic crowns fabricated with two different stereolithography systems (CeraFab 7500–CF, Alumina, multifunctional acrylate–Lithoz; CSL 150–CS, Zirconia, HDDA, PET4A–PORIMY). Both CeraFab and CSL 150 can fabricate ceramic crowns with high dimensional accuracy and marginal adaptation within clinically acceptable limits. The results indicate that the fabrication of ceramic crowns by using the SLA technique is promising.

Ioannidis et al. [49] compared the marginal and internal fit of 3D printed zirconia occlusal veneers with CAD-CAM fabricated zirconia or heat-pressed lithium disilicate ceramic (LS2) restorations on molars (ceramic powder 3 mol% yttria-stabilized zirconia polycrystal; CeraFab 7500, Lithoz GmbH). Three-dimensionally printed zirconia occlusal veneers produced using lithography-based ceramic manufacturing had a marginal adaptation (95 mm) and a production accuracy (26 mm) similar to those of conventional methods.

Canto-Naves et al. [43] investigated the internal and marginal adaptation between printed and milled onlays and found that the adaptation of the printed specimen to the prepared tooth was better than milled and that the gap reproducibility was higher.

Lyu et al. analyzed in two different papers [51,52] the dimensional accuracy of monolithic zirconia crowns fabricated with the nanoparticle jetting technique and found that this technique had better accuracy than the subtractive manufacturing and that even though printing orientation affected the accuracy of the overall, external, marginal, and intaglio regions of the crown, all printing orientation yielded values of trueness for dimensional accuracy that fulfilled the clinical requirements (<100 μm).

Shin et al. [61] investigated the effect of cement space settings on the marginal and internal fit of 3D printed definitive resin crowns and reported that the 70 μm cement gap setting had a significantly better fit in the marginal, axio-occlusal, and occlusal areas compared to the other groups. Nevertheless, all median values of the marginal gaps were within the clinically acceptable limit (<120 μm).

Suksuphan et al. [62] compared the marginal adaptation of milled and 3D printed hybrid dental crown materials with various occlusal thicknesses and found that the 3D printing technique provides better marginal adaptation than the milling one.

Zhu et al. [64] analyzed the accuracy and margin quality of 3D printed monolithic zirconia crowns and found that curved surfaces are more error-prone compared with vertical surfaces in 3D printing because of the surface stepping phenomenon. Therefore, areas like the margin or occlusal surface did not show advantages compared to the milling group. However, the axial surface had a significant advantage. They concluded that reducing the minimum layer thickness of a 3D printer is an effective method of improving its trueness.

However, as observed for the other properties studied, despite the numerous studies with positive results concerning the accuracy and fit of 3D printed materials, some authors reported unfavorable results [26,36,37].

Revilla-León et al. [26] measured and compared the marginal and internal discrepancies of milled and additively manufactured zirconia crowns (3DMix ZrO_2_ paste, 3DCeram Co.; CERAMAKER 900, 3DCeram Co.), by using the silicone replica technique. The results of the study showed that milled zirconia had clinically acceptable marginal and internal discrepancies, while the additively manufactured group had clinically unacceptable marginal and internal crown discrepancies.

Li et al. [36] analyzed the internal and marginal adaptation of SL-manufactured zirconia dental crowns from a custom-made resin-based zirconia (45 vol%; CSL 150, Porimy, Kunshan, China) and showed that SL-manufactured zirconia dental crowns have less-than-ideal internal and marginal adaptation for clinical application.

A different material was studied by Munoz et al. [37] who evaluated and compared the margin discrepancy of complete gold crowns (CGCs) fabricated from printed (ProJet DP 3000, 3D Systems), milled, and conventional hand-waxed patterns. The results showed that ProJet DP 3000 printed patterns were significantly different from LAVA CNC 500 milled and hand-waxed patterns, with an overall poorer result. Fabricating CGCs from printed patterns produced a significantly higher number of crowns with unacceptable margin discrepancies (>120 mm).

### 3.3. Aesthetic Properties

Aesthetics is another relevant aspect of permanent dental restorations, but it has not been widely studied so far. According to our findings, only a few articles [38,41,42,44,45,46] on the topic were found in the literature.

Baumgartner et al. [38] used stereolithographic ceramic manufacturing (LCM–Powder IPS e.max Press LT, Ivoclar Vivadent AG, Schaan) to reproduce lithium disilicate glass-ceramic samples, thereby demonstrating the possibility of using this technology to reproduce print dense and reliable lithium disilicate glass-ceramic samples that meet the high requirements for dental restorations regarding aesthetic properties. The printed parts’ opacity (59.9%) conforms to measurements of the powder manufacturer for pressed samples of IPS e.max Press lithium disilicate (62%). The slightly lower opacity results from smaller lithia crystal sizes compared to the pressed samples (up to 3 μm). With an optimized post-processing thermal intervention, a high level of translucency could be achieved independent of the layer thickness, resulting in more aesthetic dental restorations [38]. Espinar et al. [45,46] in two articles investigated the influence of the printing angle on the color and translucency of 3D printed resins and found a correlation. Daghrery et al. [44] and Cakmak et al. [41] investigated the color stability of 3D printed versus indirectly or subtractively fabricated veneers and found that the former were more vulnerable to discoloration and were significantly affected by artificial aging in a staining solution compared to the latter. Cakmak et al. in another article [42] found that polishing techniques influence the surface roughness and color stability of additively manufactured definitive restorations.

### 3.4. Biological Properties

The biocompatibility of 3D printed materials is a relevant aspect that must be taken into consideration. According to the current European Council Directive 93/42/EEC on medical devices, materials for short-term use in the oral cavity must meet the requirements for Class I, while for long-term use in the oral cavity, they must meet the biocompatibility requirements of Class IIa.

Despite the importance of the subject, only one study investigated the biocompatibility of 3D printable resins [56]. In that study, Nam et al. reported that surface glazing increased cell compatibility while reducing the protein adsorption of 3D printed dental resins. Thus, for 3D printed resins, a glazed surface exhibited a positive effect on those biological properties.

## 4. Discussion

This narrative review of the literature showed that crowns and partial restorations represent the large majority of the studied appliances of 3D printing materials used for the manufacture of definitive prosthodontic solutions, and the attention of investigators is primarily focused on their mechanical behavior and dimensional accuracy/fit tested in vitro. However, it was noticed that the evidence so far collected on 3D printed materials for permanent restorations is still quantitatively scarce and of limited reliability due to the huge heterogeneity of the research protocols currently adopted. This is probably because 3d printed materials for use are relatively recent materials for which there is still no consensus on the required standards for in vitro studies.

In almost all of the studies reviewed, crowns and partial restorations were printed using SLA (stereolithography), NPJ (nanoparticle jetting), and DLP (digital light processing) 3D printing technologies. The most studied materials were polymer-based composites and zirconia.

The mechanical properties of 3D printing materials, in comparison with the present materials and technologies available on the market, are widely studied. In most of the studies, 3D printed materials demonstrated their great potential to replace traditional fabrication methods. However, even if several papers reported positive results, negative results were also reported. Revilla-León et al. [34] measured significantly lower flexural strength values in comparison with the conventional milling process, and Uçar et al. [35] stated that these new materials and technologies seem to be promising but need improvements, particularly to reduce flaws. One of the main disadvantages concerning printable resins is linked to their filler volume. It has been shown that a greater amount of filler might impair the resin’s flow during the building process, therefore increasing the risk of incorporating air bubbles or areas of non-homogenous microstructure, consequently impairing mechanical properties [57]. For this reason, the printable resins currently available on the market consist of a significantly smaller amount of filler (30–50 wt%) compared to the resins designed for subtractive manufacturing (80–85 wt%). This reduced amount of filler correlates linearly with the flexural strength [68] and resulted therefore in lower values of initial strength for the 3D printed specimens compared to the milled ones, which showed higher filler load [57]. Also, zirconia seems to have lower flexural strength when 3D printed than milled, but this property was shown to be highly dependent on the building direction. When printed with an angle of 90°, the flexural strength reached the highest values, followed by the 45° and 0° printing angles [53]. Additionally, flexural strength was higher when building and loading directions were parallel compared to that of specimens in which they were perpendicular [53]. Even though the flexural strength of 3D printed zirconia was lower than milled zirconia, it still reached values of 800 MPa or higher in the 45° and 90° directions [53], being suitable for fixed dental prostheses with four or more units [69]. These findings of Miura et al. [53] conflict with Giugliano et al. [47] who, using a 0° printing angle found that 16.3% of printed specimens did not reach the 500 MPa minimum required threshold for a three-unit prosthesis. As these differences are indeed wide, this subject requires further studies, especially concerning printing orientation and its correlation with specimen testing.

In line with Ucar et al. [35], Refaie et al. [58] stated that zirconia manufacturing via 3D printing still needs improvements, especially in terms of porosity. Milled zirconia crowns showed significantly higher fracture resistance compared to the 3D printed crowns. The authors ascribed this weakness to the entrapment of air voids in the paste of the 3D printed zirconia which results in an inner porous material more prone to origin fracture. This effect has been studied in the literature, and no interlayer delamination has been found, meaning that the cracks are not related to a bad interlayer binding but rather to the porosity created by air bubble entrapping [70] and possible subsequent flaw determination. Another mechanical property widely tested among the included papers is the material’s hardness. Miura et al. [53] found that the Vickers hardness of 3D printed zirconia is similar to that of milled zirconia. In particular, the Vickers hardness of DLP-manufactured zirconia specimens was approximately 5% lower than that of subtractively manufactured specimens. Conversely, the 3D printed resins seem to have low values of microhardness. In one study [40], the nanohybrid resin tested presented values lower than that of the enamel which is therefore prone to surface wear. To enhance the microhardness of printed resins, a possible improvement seems to be surface glazing. Nam et al. [56] found that the Vickers hardness of the samples with glazed surfaces was higher than that of the samples with untreated surfaces.

Generally speaking, there is a general agreement that these new 3D materials and technologies have the potential to replace traditional fabrication methods, even if there is still a great variability of mechanical properties depending on the fabrication method and settings [38]. It is worthwhile to note that even if new 3D printed resin materials for permanent use have been marketed, little or no information is available for this category of materials, unlike CAD/CAM composite resin for permanent restorations [71,72,73].

In addition to the mentioned mechanical properties, dimensional accuracy and fit have also been investigated. As in the previous case, in most studies, 3D printed materials showed great potential to replace traditional fabrication methods. Once again, despite the numerous studies with positive results in favor of the dimensional accuracy and fit of 3D printed materials, other authors reported a clinically unacceptable marginal and internal adaptation and discrepancy. In this regard, if the proper printing settings are used and particular care is taken with the post-processing procedures, additive manufacturing overcomes some limitations of the milling technologies. The latter, in fact, due to the dimensions of the burs may have difficulties in reproducing a sharp design of the prepared tooth and may also produce marginal defects mainly due to the material chipping [43,52,62,64]. Lyu et al. [51] investigated the effect of build angle on the dimensional accuracy of monolithic zirconia crowns fabricated with the nanoparticle jetting technique and found that for incisors, a build angle of 135° yielded more accurate marginal and intaglio surfaces than other angles tested, while for molars, significantly better accuracy overall and in external and intaglio regions was found with 0° and 180° angles [51]. Their results were attributed to the smaller number of layers obtained in this direction which resulted in less residual stress caused by layer interaction in the sintering phase which can determine some deformation of the material [51].

Concerning aesthetics, Baumgartner et al. [38] showed the possibility of using this technology to reproduce print dense and reliable lithium disilicate glass-ceramic samples that meet the demanding aesthetics for dental restorations. Another paper compared the translucency of CAD/CAM resin composites for permanent use with a 3D printed resin for permanent use as well, reporting that the single opacity available for the 3D printed material showed an intermediate opacity when compared to the other CAD/CAM materials that were all marketed in two different translucencies [74]. Nevertheless, even though printable resins are marketed with only one translucency, clinicians may vary the final translucency of one restoration by managing the build angle. Espinar et al. [45] found in their study that most of the resins tested were more translucent when printed at 90°, while others were more translucent when printed at 0°. Printed specimens are composed of many layers which may have distinct refractive indices [75]. These layers and the interfaces forming the multilayer specimen are all responsible for the reflection or transmission of light [75]. The light passing through the material can be scattered or absorbed in the layers as well as transmitted or reflected at the layer’s interfaces of different refractive indices [76]. This difference in overall scattering/absorption and reflectance/transmittance values could explain the differences in translucency from the same resin depending on the printing direction. The variability of chemicals present in resins from different brands and shades could entail different scattering/absorption values in the layers and different reflectance/transmittance values at the interface of the layers and therefore different variations in translucency depending on printing orientation. This could be the reason for different magnitudes of the translucency differences due to the printing angle for different materials [45]. In the same paper, it was shown that the above-mentioned factors are also responsible for a difference in the final color of a restoration. A different printing angle produced indeed a ΔE higher than the acceptability threshold among measurements performed on the same material [45].

Concerning color stability, Daghrery et al. [44] reported that veneers manufactured using the 3D printing technique are more vulnerable to discoloration and significantly affected by artificial aging compared to indirect prefabricated veneers. This is because the color change in a resin material is highly dependent on the composition of the resin and filler content. Resin-based materials with lower filler volumes absorb more water, leading to hydrolytic degradation and ultimately to a greater susceptibility to staining [77]. Furthermore, the 3D printed material is more prone to staining due to the presence of multiple layers. The incomplete polymerization at these interfaces, along with the presence of microporosities and residual monomers, can eventually lead to a higher discoloration.

Even if biocompatibility testing is performed by the companies and revised by the competent national and supranational administrations, the biological properties of 3D printed materials for permanent restorations, despite the obvious clinical relevance, are almost completely unexplored in the scientific literature. Only one study was found investigating the biological properties of 3D printed resin materials [56]. In this study, the effect of surface glazing on cell viability was investigated. The authors found that glazing reduced the surface roughness of specimens and therefore plaque accumulation. Moreover, surface glazing interferes with protein absorption [78] by making the surface hydrophobic. Independently from the glazing, the different materials tested in the study presented various degrees of protein absorption, showing a material-dependent variability. This has been reported to be correlated to the 3D-printing-specific workflow. The 3D printed resins used in the study, following manufacturers’ instructions and a common 3D printing workflow, were post-cured in free air, that is, in the presence of oxygen, leaving an unpolymerized monomer layer on the surface. To remove it, specimens were washed in alcohol for 15 min before post-curing, but the authors reported difficulties in completely removing this unpolymerized monomer layer. The presence of these monomers affected cell viability [56]. By glazing, the leaching of residual monomers is reduced, and hence, the cell cytotoxicity is also reduced.

Other biological aspects should be further investigated like plaque accumulation, biofilm formation, and monomer leakage from the resins, both for the short and long term.

An outcome of the present review is the absence of uniformity in the research protocols, especially related to the wide differences in terms of materials, 3D printers, fabrication settings, and digital workflow. It appears that the traditional ISO standards are not yet released and/or sufficiently updated concerning these new technologies when applied to dentistry. As a result, the test standard used, as well as the methodologies shown, were not homogeneous, making direct comparison of the results across the literature challenging.

Current scientific evidence on 3D printable materials for intraoral use in Restorative and Prosthetic Dentistry concerning permanent restorations is still quantitatively and qualitatively limited. It is expected that 3D printing technology will see more widespread use in everyday clinical practice in the very near future. Therefore, the scientific evidence should be significantly consolidated, both through the definition of standards for laboratory testing to be shared by the international scientific community and by starting the necessary clinical investigations.

## Figures and Tables

**Table 1:** Search terms, search strategy, and number of documents retrieved for each database.

MEDLINE/PubMed	
Search query	(restorative dentistry OR prosthetic dentistry) AND (3D printing OR additive manufacturing OR rapid prototyping) AND materials
Search strategy	((“restorative dent”[Journal] OR (“restorative”[All Fields] AND “dentistry”[All Fields]) OR “restorative dentistry”[All Fields] OR (“prosthodontics”[MeSH Terms] OR “prosthodontics”[All Fields] OR (“prosthetic”[All Fields] AND “dentistry”[All Fields]) OR “prosthetic dentistry”[All Fields])) AND (“printing, three dimensional”[MeSH Terms] OR (“printing”[All Fields] AND “three dimensional”[All Fields]) OR “three-dimensional printing”[All Fields] OR (“3d”[All Fields] AND “printing”[All Fields]) OR “3d printing”[All Fields] OR (“addit manuf”[Journal] OR (“additive”[All Fields] AND “manufacturing”[All Fields]) OR “additive manufacturing”[All Fields]) OR ((“rapid”[All Fields] OR “rapidities”[All Fields] OR “rapidity”[All Fields] OR “rapidness”[All Fields]) AND (“prototypal”[All Fields] OR “prototype”[All Fields] OR “prototype s”[All Fields] OR “prototyped”[All Fields] OR “prototypes”[All Fields] OR “prototypic”[All Fields] OR “prototypical”[All Fields] OR “prototypicality”[All Fields] OR “prototypically”[All Fields] OR “prototyping”[All Fields]))) AND (“material”[All Fields] OR “material s”[All Fields] OR “materials”[All Fields])) AND ((y_10[Filter]) AND (fft[Filter]) AND (english[Filter]))
Retrieval	1070 items
**Scopus**	
Search query	(restorative dentistry OR prosthetic dentistry) AND (3D printing OR additive manufacturing OR rapid prototyping) AND materials
Search strategy	(restorative dentistry OR prosthetic dentistry) AND (3D printing OR additive manufacturing OR rapid prototyping) AND materials
Retrieval	908 items
**Cochrane Library**	
Search query	(restorative dentistry OR prosthetic dentistry) AND (3D printing OR additive manufacturing OR rapid prototyping) AND materials
Search strategy	((restorative dentistry OR prosthetic dentistry) AND (3D printing OR additive manufacturing OR rapid prototyping) AND materials) in Title Abstract Keyword
Retrieval	123 items
**Web of Science**	
Search query	(restorative dentistry OR prosthetic dentistry) AND (3D printing OR additive manufacturing OR rapid prototyping) AND materials
Search strategy	(restorative dentistry OR prosthetic dentistry) AND (3D printing OR additive manufacturing OR rapid prototyping) AND materials
Retrieval	367 items

**Table 2:** Included studies and assessed variables.

Study	Objective (S)	Material and 3D Printer	Mat.Type	Evaluated Parameter (S)	Main Conclusion (S)
Bae et al., 2017 [27]	To evaluate the accuracy of inlay restorations fabricated by AM compared to subtractive methods.	Polymer (VisiJet FTX Green, 3D Systems); 3D Printer (ProJet 1200–3D Systems).	P	Dimensional accuracy and surface morphology.	The accuracy of inlays fabricated by AM is higher than that of subtractive methods.
Baumgartner et al., 2020 [38]	To use stereolithographic ceramic manufacturing (LCM) to reproducibly print dense and reliable lithium disilicate glass-ceramic samples.	Powder (IPS e.max Press LT, Ivoclar Vivadent AG, Schaan).	C	Mechanical properties and aesthetics.	It is possible to use stereolithographic ceramic manufacturing (LCM) to reproducibly print dense and reliable lithium disilicate glass-ceramic samples that meet the high requirements for dental restorations regarding mechanical properties and aesthetics.
Borella et al., 2023 [40]	To evaluate the physical and mechanical properties of four 3D printed resins with two different thickness layers.	Polymer (VarseoSmile Crown Plus, VSC); 3D printer (Anycubic Photon Mono, Anycubic 3D).	P	Raman spectroscopy for degree of conversion, confocal laser scanning microscopy for surface roughness (Sa), three-point bending test for flexural strength and elastic modulus, and a Vickers hardness test (VHN).	The physical and mechanical properties of three-dimensional printed restorations can be affected by the layer thickness, which can interfere with the choice of the 3D printing resin for a desired clinical outcome.
Cakmak et al., 2023 [41]	To evaluate the surface roughness, optical properties, and microhardness of additively or subtractively manufactured CAD-CAM materials after simulated brushing and coffee thermal cycling.	Polymers (Crowntec, CT and VarseoSmile Crown Plus, VS); 3D printers (MAX UV; Asiga).	P	Surface roughness, Vickers microhardness, and color coordinates.	Tested additively manufactured resins can be considered more susceptible to simulated brushing and coffee thermal cycling than the other materials, given the fact that their surface roughness and ΔE00 values were higher than previously”reported acceptability thresholds and because they had the lowest microhardness after all procedures were complete.
Cakmak et al., 2023 [42]	To evaluate how different polishing techniques and coffee thermal cycling affect the surface roughness and stainability of additively and subtractively manufactured resins used for definitive prostheses.	Polymers (Crowntec, CT and VarseoSmile Crown Plus, VS); 3D printers (MAX UV; Asiga).	P	Surface roughness and color stability.	R(a) of CS was similar to or lower than the R(a) of other materials, regardless of the time interval or polishing technique. CP mostly led to lower R(a) than other polishing techniques, whereas VA resulted in a high R(a) regardless of the material–time interval pair. Polishing reduced the R(a), while coffee thermal cycling was found to have a small effect. Among tested material-polishing pairs, only CS-VA had moderately unacceptable color change when previously reported threshold values were considered.
Canto-Naves et al., 2023 [43]	To compare the gaps between the prepared tooth and milled and printed onlays fabricated with the same CAD design. It also aimed to determine the gap reproducibility across onlays fabricated by 3D printing and milling.	Polymer (Permanent Crown Resin A2; Bego GmbH); 3D printer (Formlabs Form 3+).	P	Internal and marginal adaptation.	This study concluded that the printed onlays adapted significantly better to the prepared tooth than the milled onlays. Printed onlays also showed significantly better gap reproducibility.
Daghrery et al., 2023 [44]	To evaluate the effect of artificial aging by immersion in different staining solutions on the color changes, gloss, and surface roughness (Ra) of 3D printed veneers compared to the prefabricated resin composite veneer systems (PRCVs) manufactured by Componeer and Edelweiss.	Polymers (Iris Max DWS, Componeer Brilliant); 3D printer (DFAB Chairside 3D blue edge laser printer).	P	Color stability, gloss retention, and surface roughness.	Veneers manufactured using the 3D printing technique are vulnerable to discoloration and are significantly affected by artificial aging in a staining solution compared to the PRCVs. Coffee and tea staining had a deleterious effect on the color, surface gloss, and surface roughness of all tested indirect composite veneers despite manufacturing techniques. The efficacy of stain removal was higher with an in-office bleaching technique compared to surface polishing in the PRCVs, while in-office bleaching and surface polishing showed comparable effects in the 3D printed veneers. Veneer production using 3D printing provides cost-effective, time-efficient, and on-demand solutions. However, material processing for 3D printing is crucial for long- term longevity.
Espinar et al., 2023 [45]	To evaluate the influence of printing orientation on color and translucency of 3D printing restorative resins.	Polymer (FP-Formlabs Permanent Crown); 3D printer (3D Form 3B+).	P	Color and translucency.	The selection of building orientation (0 degrees and 90 degrees) for the 3D printed resins influences the visual color and translucency and therefore their esthetic appearance.
Espinar et al., 2023 [46]	To evaluate the influence of thickness and printing angle on the optical properties of 3D printed dental restorative resins.	Polymer (FP-Formlabs Permanent Crown); 3D printer (3D Form 3B+).	P	Scattering (S), absorption (K) and albedo (a) coefficients, transmittance (T%), light reflectivity (RI), and infinite optical thickness (X infinity).	Optical properties of 3D printed restorative resins vary between thicknesses and could be affected by the building orientation.
Giugliano et al., 2023 [47]	To compare the dimensional accuracy, translucency, and biaxial flexural strength of milled zirconia (MZ) versus 3D printed zirconia (PZ) discs.	Zirconia (LithaCon 3Y 230, Lithoz America); 3D printer (Lithoz Cerafab 7500 Dental 3D-printer).	C	Translucency, flexural strength.	The results showed that the milled specimens achieved better dimensional accuracy and were more translucent, stronger, and less prone to failure than printed specimens.
Homsy et al., 2018 [48]	To compare the marginal and internal fit accuracy of lithium disilicate glass-ceramic inlays fabricated with conventional, milled, and three-dimensional (3D) printed wax patterns.	Polymer (VisiJet FTX Green, 3D Systems); 3D Printer (ProJet 1200–3D Systems).	P	Marginal and internal fit accuracy.	The CAD-CAM subtractive method of wax pattern fabrication produced IPS e.max. Press inlays with better marginal and internal fittings than those obtained through conventional workflow or additive 3D printing. Three-dimensional printing of inlay wax patterns yielded similar results to conventional waxing in terms of marginal and internal fit.
Ioannidis et al., 2022 [49]	To compare the marginal and internal fit of 3D printed zirconia occlusal veneers with CAD-CAM fabricated zirconia or heat-pressed lithium disilicate ceramic (LS2) restorations on molars.	Material (ceramic powder 3 mol% yttria-stabilized zirconia polycrystal); 3D Printer (CeraFab 7500; Lithoz GmbH).	C	Marginal and internal fit.	Three-dimensionally printed zirconia occlusal veneers produced by means of lithography-based ceramic manufacturing exhibit a marginal adaptation (95 mm) and a production accuracy (26 mm) similar to those of conventional methods.
Karaoglanoglu et al., 2023 [50]	To investigate the surface roughness, microhardness, and color changes of resin-based computer-aided design/computer-aided manufacturing (CAD/CAM) blocks and 3D printed permanent resins in different beverages.	Polymer (Crowntec and Permanent Crown); 3D printer (MAX UV; Asiga).	P	Surface roughness, microhardness, and color.	Although the surface roughness of 3D printed permanent resins was similar to that of resin-based CAD/CAM blocks, they had a lower microhardness value. Moreover, 3D printed permanent resins showed more color changes in tea and coffee.
Li et al., 2019 [36]	To evaluate the physical and mechanical properties of SL-manufactured zirconia dental crowns and analyze their internal and marginal adaptation.	Custom-made resin-based zirconia (45 vol%); 3D Printer (CSL 150–Porimy).	C	Density, sintering shrinkage, flexural strength, Weibull parameters, internal marginal adaptation.	The strength of SL-manufactured zirconia was adequate to fabricate dental crowns, which showed less-than-ideal internal and marginal adaptation for clinical applications.
Lyu et al., 2023 [51]	To evaluate the effect of the build angle on the dimensional accuracy of monolithic zirconia complete crowns fabricated by using NPJ.	Zirconia (C800 Xjet); 3D printer (Carmel 1400C Xjet).		Dimensional accuracies in the external, marginal, and intaglio regions.	The dimensional accuracy of monolithic zirconia crowns fabricated by using NPJ was affected by the build angle and was within clinically acceptable limits.
Lyu et al., 2023 [52]	To compare the dimensional accuracy and clinical adaptation of zirconia crowns fabricated with NPJ and those fabricated with subtractive manufacturing (SM) and digital light processing (DLP).	Zirconia (C800 Xjet); 3D printer (Carmel 1400C Xjet).	C	Dimensional accuracy in the external, intaglio, and marginal areas,	Monolithic zirconia crowns fabricated using NPJ have higher dimensional accuracy and clinical adaptation than those fabricated using SM or DLP.
Miura et al., 2023 [53]	To evaluate the mechanical and surface properties of zirconia manufactured using additive manufacturing (AM) technology and the effect of the building direction on the mechanical and surface properties.	Zirconia (3Dmix ZrO(2) 3Dceram); 3D printer (CeraMaker 900 3D Ceram).	C	Flexural strength, Vickers hardness, fracture toughness, elastic modulus, and Poisson’s ratio.	The flexural strength and surface structure of the tested SLA-manufactured zirconia were influenced by the building direction; however, other mechanical properties remained unaffected. The layer boundaries affected the anisotropic behavior of the builds to a certain extent, owing to the layer-by-layer production method.
Miura et al., 2023 [54]	To investigate the effect of low-temperature degradation (LTD) on the mechanical properties of additive-manufactured zirconia.	Zirconia (3Dmix ZrO(2) 3Dceram); 3D printer (CeraMaker 900 3Dceram).	C	Flexural strength, modulus of elasticity, Vickers hardness, and fracture toughness.	High average material strengths that exceed the current ISO requirements for fixed ceramic prostheses were measured in flexural tests, except in the 0° direction. LTD had little effect on flexural strength, elastic modulus, Vickers hardness, and fracture toughness. However, the optimization of all processing steps, including 3D printing, cleaning, stripping, sintering, and color immersion, is necessary to achieve optimal reliability of 3D printed zirconia materials for clinical applications.
Munoz et al., 2017 [37]	To evaluate and compare margin discrepancy of complete gold crowns (CGCs) fabricated from printed, milled, and conventional hand-waxed patterns.	Material (Gold); 3D printer (ProJet DP 3000; 3D Systems).	M	Margin discrepancy.	The ProJet DP 3000 printed patterns were significantly different from LAVA CNC 500 milled and hand-waxed patterns, with an overall poorer result. Fabricating CGCs from printed patterns produced a significantly higher number of crowns with unacceptable margin discrepancy (>120 mm).
Nakai et al., 2021 [55]	To assess the crystallography, microstructure, and flexural strength of zirconia-based ceramics made by stereolithography (SLA).	Two additively manufactured 3Y-TZPs (LithaCon 3Y 230, Lithoz, Vienna, Austria; 3D Mix zirconia, 3Dceram Sinto, Limoges, France), one additively manufactured ATZ (3D Mix ATZ, 3Dceram Sinto, Limoges, France); 3D Printer (SLA).	C	Crystallography, microstructure, and flexural strength.	Additively manufactured zirconia revealed a crystal structure, biaxial flexural strength, and microstructure comparable to that of subtractively (conventionally) manufactured zirconia. Differences in the additive manufacturing process of zirconia may affect the biaxial flexural strength of additively manufactured zirconia. Additively manufactured ATZ had a higher biaxial flexural strength than additively and subtractively manufactured 3Y-TZP.
Nam et al., 2023 [56]	To determine the surface glazing effect on the mechanical and biological properties of three-dimensional printed dental permanent resins.	Polymer (Formlabs, Graphy Tera Harz permanent); 3D printer (Next Dent 5100).	P	Flexural strength, Vickers hardness, color stability, and surface roughness.	Surface glazing increased the mechanical strength, color stability, and cell compatibility while reducing the Ra and protein adsorption of 3D printed dental resins. Thus, a glazed surface exhibited a positive effect on the mechanical and biological properties of 3D printed resins.
Prause et al., 2023 [57]	To evaluate the flexural strength and fatigue behavior of a novel 3D printed composite resin for definitive restorations.	Polymer (Varseo Smile Crown Plus); 3D printer (Varseo XS).	P	Biaxial flexural strength and biaxial flexural fatigue strength.	The 3D printed composite resin exhibited the lowest mechanical properties, where areas of nonhomogeneous microstructure developed during the mixing procedure served as potential fracture origins.
Refaie et al., 2023 [58]	To evaluate the effect of cyclic mechanical loading on the fracture resistance of 3D printed zirconia crowns in comparison to milled zirconia crowns.	Zirconia (Lithoz 210 3Y); 3D printer (Cera Fab7500).	C	Fracture resistance.	The fabrication technique and cyclic loading affect the fracture resistance of zirconia crowns. Although the fracture resistance values for the 3D printed crowns were lower than those of the milled ones, they are higher than the masticatory forces and thus could be considered clinically acceptable.
Revilla-Leon et al., 2020 [26]	To measure and compare the marginal and internal discrepancies of milled and AM zirconia crowns by using the silicone replica technique.	Zirconia (3Dmix ZrO_2_ paste; 3Dceram Co.); 3D Printer (CERAMAKER 900; 3Dceram Co.).	C	Marginal and internal discrepancies.	CNC and SAM groups had clinically acceptable marginal and internal discrepancies, while the AM group had clinically unacceptable marginal and internal crown discrepancies.
Revilla-Leon et al., 2021 [34]	To compare the flexural strength and Weibull characteristics of milled and additively manufactured zirconia.	Photosensitive resin mixed with zirconia paste (3DmixZrO_2_L, 3Dceram Co.); 3D Printer (CERAMAKER 900; 3Dceram Co).	C, P	Flexural strength and Weibull characteristics.	AM zirconia material revealed significantly lower flexural strength mean values than milled zirconia material. Significantly decreased flexural strength values of milled and AM zirconia material as indicated by the Weibull. Moduli were significantly higher for the milled groups than the additively manufactured groups.
Revilla-Leon et al., 2022 [59]	To measure the manufacturing accuracy and volumetric changes of additively manufactured (AM) zirconia specimens with different porosities (0%, 20%, and 40%).	Material (3Dmix ZrO_2_ paste; 3Dceram Co.); 3D Printer (CERAMAKER 900; 3Dceram Co.).	C	Manufacturing accuracy and volumetric changes.	The 40%-porosity group obtained the highest manufacturing accuracy and the lowest manufacturing volume change, followed by the 20%-porosity and the 0%-porosity groups. An uneven manufacturing volume change in the x-, y-, and z-axes was observed. However, none of the groups tested were able to perfectly match the virtual design of the specimens.
Rosentritt et al., 2023 [60]	To compare the in vitro performance and wear behavior of additively or subtractively fabricated resin-based composite molar crowns for temporary and permanent application.	Polymer (VarseoSmile Crown plus); 3D printers (Varseo XS, Asiga MAX UV).	P	Fracture force, wear, and roughness.	Temporary and permanent molar crowns provided at least acceptable in vitro performance and fracture force for clinical mid-term application. Laboratory wear stability of the resin-based materials appeared sufficient but should be verified under clinical conditions.
Shin et al., 2023 [61]	To evaluate how cement gap settings affect the marginal and internal fit of a 3D printed definitive resin crown.	Polymer (TC-80DP); 3D printer (Sprint Ray Pro 95).	P	Marginal and internal fit.	Based on the findings of this in vitro study, a 70 μm cement gap setting is recommended for optimal marginal and internal fit of 3D printed resin crowns.
Suksuphan., 2023 [62]	To evaluate the marginal adaptation and fracture resistance of three computer-aided design/computer-assisted manufacturing hybrid dental materials with different occlusal thicknesses.	Polymer (Varseosmile Crown Plus); 3D printer (Freeform Pro 2).	P	Marginal adaptation and fracture resistance.	All hybrid-material crowns demonstrated favorable marginal adaptation within a clinically acceptable range, with 3D printing yielding superior results to milling. All materials could withstand normal occlusal force even with a 0.8 mm occlusal thickness.
Ucar et al., 2019 [35]	To compare the mechanical and microstructural properties of ceramics from lithography-based ceramic manufacturing (LCM) with pressing and CAD/CAM methods.	High-purity alumina (LithaLox HP 500, Lithoz); 3D Printer (CeraFab 7500, Lithoz).	C	Biaxial flexural strength, hardness, fracture toughness, structural reliability.	LCM can be used to produce ceramic parts. Mechanical properties and manufacturing of LCM ceramics seem to be promising but need improvements, mainly to reduce porosity.
Wang et al., 2019 [63]	To evaluate the trueness of zirconia crowns fabricated by 3D printing in comparison with crowns fabricated by CAD-CAM milling as a control.	Photosensitive resin mixed with zirconia paste (3DmixZrO2L, 3Dceram Co.); 3D Printer (CERAMAKER 900; 3Dceram Co.).	C, P	Trueness (dimensional accuracy considering 4 crown locations).	Zirconia crowns produced by 3D printing met the trueness requirements, and 3D printing may be suitable for fabricating zirconia crowns.
Wang et al., 2021 [29]	To evaluate the dimensional accuracy and clinical adaptation of ceramic crowns fabricated with the stereolithography technique.	Material (Alumina, multifunctional acrylate–Lithoz; Zirconia, HDDA, PET4A–PORIMY); 3D Printer (CeraFab 7500, Lithoz).	C, P	Dimensional accuracy and marginal adaptation.	Both CF and CL can fabricate ceramic crowns with high dimensional accuracy and marginal adaptation within clinically acceptable limits. The results indicated that the fabrication of ceramic crowns by using the SLA technique is promising.
Zandinejad et al., 2019 [28]	To compare the fracture resistance of milled zirconia (MZr), milled lithium disilicate (MLD), and AM zirconia (AMZr) crowns when cemented to MZr implant abutment.	Composite (3Dceram Co. Lemonge, France); 3D Printer; (CeraMaker 900; 3Dceram Co. Lemonge, France).	P	Fracture load.	AM all ceramic crowns cemented on zirconia abutments had a comparable fracture resistance to milled restorations in this in vitro study. AM appears to be a promising technology for the fabrication of all ceramic restorations with great potential for improvement in the near future.
Zhu 2023 [64]	To evaluate and compare the trueness, crown fit, and margin quality of monolithic zirconia crowns manufactured by NPJ with those milled by a computer numerical control system.	Zirconia (brand not available)	C	Trueness, crown fit, and margin quality.	All 3 manufacturing methods can fabricate zirconia crowns with a clinically acceptable crown fit. The NPJ system could be used to manufacture monolithic zirconia crowns with better margin quality and proximal surface trueness than milled crowns.
Zimmermann et al., 2019 [65]	To evaluate the fracture behavior of different CAD/CAM ceramics and composites and one 3D printed composite as a function of different crown thicknesses (0.5, 1, and 1.5 mm).	Composite (els-3D Harz, Saremco Dental AG); 3D Printer (DLP Freeform Pro 2–ASIGA).	P	Fatigue and fracture load.	As none of the 0.5 mm ceramic crowns survived fatigue testing and all 0.5 mm composite crowns did, composites may have advantageous material characteristics compared to ceramic CAD/CAM materials for minimal restoration thicknesses.

C = Ceramic, P = Polymers, M = Metals.
